# Low expression of H3K27me3 is associated with poor prognosis in conventional chordoma

**DOI:** 10.3389/fonc.2022.1048482

**Published:** 2022-12-19

**Authors:** Jie Wei, Jianfeng Wu, Zhiyong Yin, Xia Li, Yixiong Liu, Yingmei Wang, Zhe Wang, Chao Xu, Linni Fan

**Affiliations:** ^1^ State Key Laboratory of Cancer Biology, Department of Pathology, The First Affiliated Hospital of Air Force Military Medical University, Xi’an, China; ^2^ Department of Cardiology, The First Affiliated Hospital of Air Force Military Medical University, Xi’an, China; ^3^ Department of Knee Joint Surgery, Honghui Hospital, Xi’an Jiaotong University, Xi’an, China; ^4^ Department of Health Statistics, Faculty of Preventive Medicine, the Air Force Military Medical University, Xi’an, China

**Keywords:** chordoma, H3K27me3, prognosis, recurrence, brachyury

## Abstract

**Purpose:**

Chordoma is a rare and locally invasive neoplasm, and the prognostic factors are limited. Deregulation of Histone 3 lysine 27 (H3K27) trimethylation (H3K27me3) is considered to be related with poor prognosis in some tumors. The purpose of this study was to detect the expression of H3K27me3 in chordomas and analyze the correlation with clinicopathological features and explore the roles as potential prognostic markers and therapeutic targets.

**Material and method:**

Specimens of 162 chordoma patients (consisting of 156 conventional chordoma, 4 dedifferentiated chordoma and 2 poorly differentiated chordoma) were enrolled in a tissue microarray (TMA) in order to assess the immunohistochemical staining by H3K27me3 antibodies. Correlations between H3K27me3 expression and clinicopathological features were analyzed. Clinical data of the patients were correlated and survival analysis was performed. *Kaplan-Meier* survival curves and *log-rank* test were used to analyze the recurrence-free survival (RFS) and overall survival (OS). Multivariate *Cox* regression analyses were used to identify potential prognostic factors.

**Results:**

The expression of H3K27me3 was lower in 37 chordoma patients (37/162, 22.8%), and higher in 125 patients (125/162, 77.2%). H3K27me3-low expression significantly correlated with spine location (*P* < 0.001), conventional histological subtype (*P* < 0.001), and recurrence (*P* < 0.001). *Log-rank* test showed that H3K27me3-low expression was associated with poor RFS (*P* =0.027) and OS (*P* =0.009) in conventional chordoma patients. *Cox* multivariate analysis revealed that low expression of H3K27me3 was an independent predictor of poor OS (*P* =0.007) and RFS (*P* =0.025) in conventional chordoma patients.

**Conclusions:**

Our study indicates that low expression of H3K27me3 might be considered as a predictor for poor prognosis and recurrence, and it may provide a potential therapeutic target for conventional chordoma patients.

## 1 Introduction

Chordomas are rare malignant bone tumors that arise from the notochordal remnants with an incidence of 0.05/100,000 people per year (1), mainly distributed in the axial bones of the human body. According to the 5^th^ edition of the World Health Organization bone and soft-tissue tumor classification, chordomas were divided into three types (conventional, dedifferentiated and poorly differentiated chordoma), of which the latter two have poorer prognosis. Due to their proximity to critical neurovascular structures, a radical surgical resection is challenging (2). Current management of chordoma relies heavily on surgery and radiation therapy, with a limited role for molecular targeted therapies such as imatinib and erlotinib (3). The 5-year overall survival for chordoma patients is 67.6% (4). Up to now, the recognized prognostic markers and potential therapeutic targets are very limited for chordoma patients, especially those with conventional chordoma (5–8). Thus, it is necessary to explore new and helpful prognostic and therapeutic markers for chordomas.

Histone 3 lysine 27 trimethylation (H3K27me3) is the tri-methylation modification on histone H3 lysine 27 and can be catalyzed by H3K27 demethylases UTX and JMJD3. It is reported that down-regulation or complete loss of H3K27me3 protein expression can be used as a specific diagnostic marker for a variety of tumors. Loss of H3K27me3 expression was found in 51% of malignant peripheral nerve sheath tumors (MPNSTs) (9). H3K27me3 staining was globally reduced in group-A childhood posterior fossa ependymoma, which was used to guide clinical treatment and prognosis (10). H3K27me3 can also assist in the diagnosis of diffuse midline glioma, which is an infiltrative midline glioma with loss of H3K27me3 (11). At the same time, some studies have found that the expression of H3K27me3 is related to poor prognosis of patients, such as synovial sarcoma, breast cancer, and anaplastic meningioma (12–14). Recently, Lucia Cottone et al. (15) found that the demethylases inhibition of the histone 3 lysine 27 targets T-box transcription factor T (TBXT, making a protein called brachyury), which is a specific marker for the notochord and induces chordoma cell death (16). This indicated that H3K27me3 might play an important role in the epigenic regulation of brachyury, thus affecting the occurrence and progression of chordoma. Our study aims to investigate, for the first time, the immunoexpression patterns of H3K27me3 in chordoma tissue samples and their correlations with clinicopathologic features and prognosis of chordoma patients, exploring the potential of H3K27me3 as a meaningfully prognostic marker and therapeutic target for chordoma patients.

## 2 Materials and methods

### 2.1 Patients

Patients presenting undergoing surgical resection of chordoma from January 2010 to December 2020 were retrospectively retrieved from the pathology databases of Xi Jing Hospital in Xi’an, China. There are a total of 266 cases. Considering that the molecular biology of relapsed patients may change, we only selected specimens from cases presented in the initial biopsy, a total of 162 specimens (consisting of 156 conventional chordoma, 4 dedifferentiated chordoma and 2 poorly differentiated chordoma). 162 chordoma patient specimens in formalin fixed paraffin embedded (FFPE) blocks obtained were used to construct the tissue microarray (TMA). The diagnoses were reviewed by three pathologists according to the 2020 WHO Classification of Tumors of Soft Tissue. The recurrence or progression of the primary tumor at the same surgical site, and/or the spread of the tumor from the primary site to the adjacent area is defined as recurrence (17). This study was approved by Ethics Committees of Xi Jing Hospital in Xi’an, China.

### 2.2 Immunohistochemical staining and scoring

A tissue microarray was produced using 4.0-mm diameter tumor cores. Immunohistochemistry staining was carried out using a BOND-MAX automated immunostainer (VisioncBioSystems) and a polymer-based detection system. IHC staining was carried out using a BOND-MAX automated immunostainer (Vision BioSystems, Leica, Victoria, Australia) and a polymer-based detection system. The primary antibodies were antibodies against H3K27me3 (Clone C36B11, Cell Signaling Technology, Danvers, MA). Staining was evaluated by 3 independent experienced pathologists who were blinded to all data according to the Immune-Reactive-Score (IRS) described by Remmele et al. in 1987, which takes into account both the percentage of positive cells and coloring strength (12). The coloring strength was classified into groups from 0 to 3 as follows: 0, no staining reaction; 1, weak staining; 2, moderate staining; and 3, strong staining. The percentage of positive cells was similarly classified from 0 to 4 as follows: 0, 0% stained cells; 1, ≤10% stained cells; 2, ≤50% stained cells; 3, 51-80% stained cells; and 4, 81-100% stained cells. The multiplication of the staining percentage and the intensity is the final immunohistochemical score (range 0-12). At an IRS of 0–3 the staining is low expression, and ≥4 is high expression.

### 2.3 Statistical analysis

All data were statistically analyzed by IBM-SPSS Statistics version 20 (IBM). For correlation analysis, the *Pearson’s chi-square* test was used. The *Kaplan-Meier* method was applied with the *log-rank* test to analyze survival. Univariate and multivariate analysis was conducted using *Cox* proportional hazards regression analysis. For *Cox* multivariate analyses, variables with *P* value <0.20 on univariate analyses were integrated into the model. As for the survival analysis, Overall survival (OS) was identified as the interval from the initial diagnosis to death of the patient. Relapse free survival (RFS) was defined as the interval from the date of first diagnosis to the earliest date of first disease recurrence or death if no recurrence is observed. A *P* value less than 0.05 was considered significant.

## 3 Results

### 3.1 Clinical data

162 patients (96 males and 66 females) were enrolled in our study, with the median age of 50 years (ranged 1-82 years). Among all cases, 93 cases were located in the skull base and the other 69 cases were in the spine. Follow-up data (up to 134 months) was available for 145 patients. The mean follow up was 51.8 ± 35.0 (mean ± standard deviation) months (ranged 1-134 months). 42.76% (62/145) had one or more recurrences (2 to 4 recurrences, respectively in 7, 4 and 1 patients), while the other 50 patients had one recurrence. The 5-year overall survival estimate was 75.9% (95% CI: 66.7% - 85.1%).

### 3.2 Histopathological evaluation

**Figure 1:**
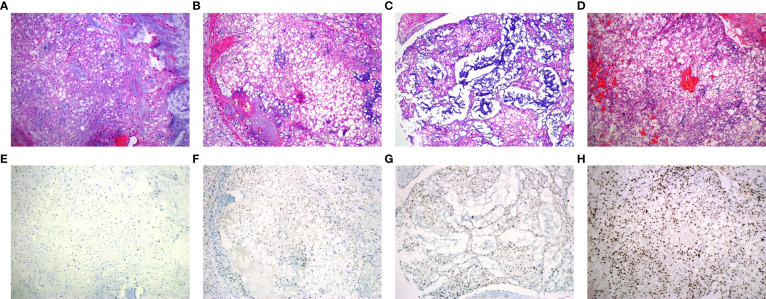
Representative patterns of H3K27me3 staining in conventional chordoma tissue microarray. **(A-D)** HE stained tissue sections. **(E, F)** Low expression of H3K27me3 in conventional chordoma. **(E)** All tumor cells with no staining, **(F)** 10% tumor cells with moderate staining. **(G, H)** High expression of H3K27me3 in conventional chordoma. **(G)** 70% tumor cells with moderate staining, **(H)** all tumor cells with distinct and strong brown staining.

**Figure 2:**
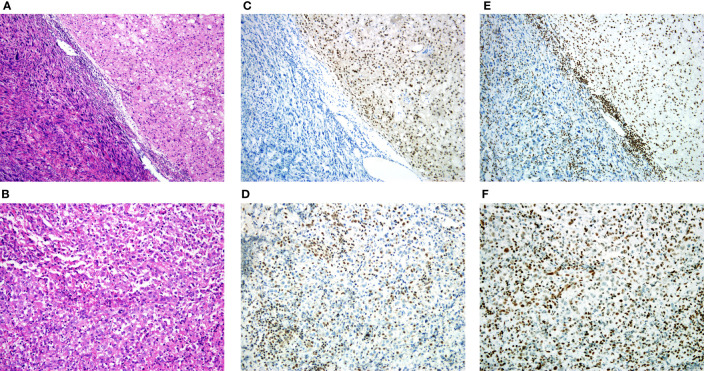
H3K27me3 expression in dedifferentiated chordoma and poorly differentiated chordoma tissues. **(A)** Dedifferentiated chordoma with typical features of conventional chordoma (right), and a transition to a high-grade pleomorphic sarcoma (left). **(B)** Poorly differentiated chordoma. **(C)** IHC staining of brachyury, noting that the dedifferentiated component did not express brachyury. **(D)** IHC staining of SMARCB1 (INI1), and the poorly differentiated chordoma showed loss of SMARCB1 (INI1). **(E, F)** IHC staining of H3K27me3, **(E)** Low expression of H3K27me3 in dedifferentiated component, **(F)** High expression of H3K27me3 in poorly differentiated chordoma.

According to the WHO classification, the cohort was consisted of 156 conventional, 4 dedifferentiated and 2 poorly differentiated chordomas. The morphology of conventional chordoma presented with a mucinous background (**Figures 1A–D**). The tumor cells were scattered or arranged in cords and nests with bubbly cytoplasm, embedded within an abundant extracellular myxoid matrix. The cases with dedifferentiated histology presented marked atypical and pleomorphism features such as sarcomatoid areas, high nuclear/cytoplasmic ratios and irregular nuclear membranes (**Figure 2A**). The cases with poorly differentiated chordoma were composed of nests or cohesive sheets of epithelioid cells. The two dedifferentiated chordomas had relatively abundant eosinophilic cytoplasm and scattered cytoplasmic vacuoles. The nuclei were round to ovoid, with vesicular chromatin, and they revealed mild to moderate pleomorphism. Extracellular myxoid stroma was not obvious (**Figure 2B**). The dedifferentiated component did not express brachyury (**Figure 2C**). Poorly differentiated chordoma showed loss expression of SMARCB1 (INI1) (**Figure 2D**). In dedifferentiated chordoma, the expression of H3K27me3 was low in dedifferentiated component while high in conventional component (**Figure 2E**). H3K27me3 was highly expressed in both poorly differentiated chordoma cases (**Figure 2F**).

### 3.3 Correlation of H3K27me3 with clinicopathological characteristics

Of 162 chordoma patients, H3K27me3 expression was low in 22.8% (37 of 162) (**Figures 1E, F**), and high in 77.2% (125 of 162) of cases (**Figures 1G, H**). Statistically, H3K27me3-low expression significantly correlated with chordoma of the spine (*P* < 0.001), recurrence (*P* < 0.001) and conventional subtype (*P* < 0.001) (**Table 1**).

**Table 1:** Characteristics of patients with chordoma.

Clinicopathological features	Total,n	H3k27me3 expression		
		Low (n,%)	High (n,%)	*P_p_* value
All patients	162	37 (22.8)	125 (77.2)
Age (years)			
<50	69	18 (26.1)	51 (74.0)	0.396
≥50	93	19 (20.4)	74 (79.6)	
Gender				
Male	96	26 (27.1)	70 (72.9)	0.121
Female	66	11 (16.7)	55 (83.3)	
Tumour location				
Skull base	93	12 (12.9)	81 (87.1)	<0.001*
Spine	69	25 (36.2)	44 (63.8)	
Recurrence				
Absent	83	12 (14.5)	71 (85.5)	<0.001*
Present	62	25 (40.3)	37 (59.7)	
Histological subtype				
Conventional	156	37 (23.7)	119 (76.3)	<0.001*
Dedifferentiated	4	0 (0)	4 (100)	
Poorly differentiated	2	0 (0)	2 (100)	

*Statistical significance (P<0.05).

**P_p_** p value for Pearson chi-square test.

### 3.4 Survival analyses

**Figure 3:**
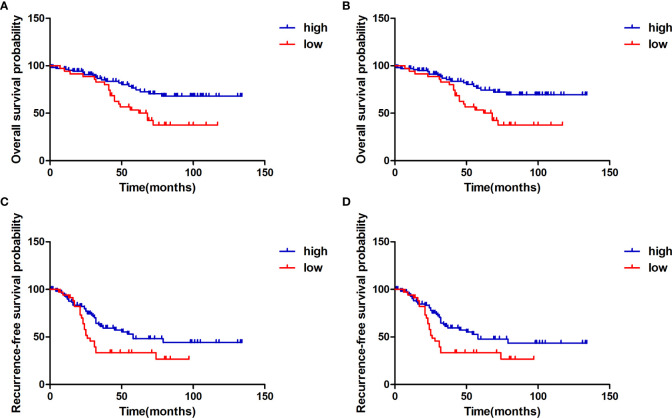
*Kaplan-Meier* survival curve with *log-rank* test derived results of survival analysis. **(A)** Overall survival of 145 chordoma patients stratifed by the expression status of H3K27me3 in all patients, **(B)** Overall survival of 139 conventional chordoma patients stratifed by the expression level of H3K27me3. **(C)** Recurrence-free survival of 145 chordoma patients stratifed by the expression level of H3K27me3 in all patients, **(D)** Recurrence-free survival of 139 conventional chordoma patients stratifed by H3K27me3 status. Compared to the high expression group, patients with low H3K27me3 expression had significantly shorter overall survival and recurrence-free survival.

To identify whether H3K27me3 expression correlates to the prognosis of chordoma patients, survival analysis was performed on OS, and RFS of the 145 chordoma patients. The mean OS in months was 51.75 (95% CI: 46.01 to 57.49), mean RFS in months was 36.23 (95% CI: 31.17 to 41.28). Univariate analysis revealed that low expression of H3K27me3 (*P* = 0.009) was significantly associated with shorter OS in 145 chordoma patients (**Table 2**; **Figure 3A**) and in 139 patients with conventional chordoma (**Figure 3B**). The low expression of H3K27me3 (*P* = 0.027) was still significantly associated with shorter RFS in 145 patients with chordoma (**Table 3**; **Figure 3C**), and in 139 patients with conventional chordoma (**Figure 3D**). Cox multivariate analysis revealed that the low expression of H3K27me3 was an independent predictor for poor OS (HR=2.235, 95% CI: 0.227-0.786, *P*=0.007, **Table 2**) and RFS (HR=1.808, 95% CI: 0.330-0.927, *P*=0.025, **Table 3**). Regarding the 4 cases with dedifferentiated histology, two of them had been nonrecurrent after being followed for 35 and 118 months. Another patient relapsed at 16 months of follow-up and died at 63 months. The remaining patient was now in 7th month of follow-up, and had developed lung metastasis. Two patients with poorly differentiated chordoma, one of them relapsed after 19 months of follow-up, and the other died of the disease.

**Table 2:** Univariate and multivariate analysis of overall survival for chordoma patients.

Variable	parameter	Univiariate analysis			Multivariate analysis		
		HR	95% CI	*P*_l_ value	HR	95% CI	*P*_c_ value
Age (years)	<50	1.730		0.079	1.713		0.105
	≥50	1	0.314-1.066		1	0.318-1.115	
Gender	Male	1.055		0.864			
	Female	1	0.570-1.955				
Tumour location	Spine	1.053		0.740			
	Skull	1	0.776-1.430				
Recurrence	Present	1.598		0.147			
	Absent	1	0.848-3.012				
H3K27me3 expression	Low expression	2.252		0.009*	2.235		0.007*
	High expression	1	0.242-0.815		1	0.227-0.786	

*Statistical significance (P<0.05).

***P_l_*** *p* value for *Log Rank* test.

***P_c_*** *p* value for *Cox* proportional hazards regression analysis.

**Table 3:** Univariate and multivariate analysis of recurrence-free survival for chordoma patients.

Variable	parameter	Univiariate analysis			Multivariate analysis		
		HR	95% CI	*P*_l_ value	HR	95% CI	*P*_c_ value
Age (years)	<50	1.392		0.193	1.410		0.177
	≥50	1	0.436-1.182		1	0.431-1.168	
Gender	Male	1.002		0.994			
	Femal	1	0.777-1.284				
Tumour location	Spine	1.155		0.261			
	Skull	1	0.898-1.485				
H3K27me3 expression	Low expression	1.792		0.027*	1.808		0.025*
	High expression	1	0.333-0.935		1	0.330-0.927	

*Statistical significance (P<0.05).

***P_l_*** *p* value for *Log Rank* test.

***P_c_*** *p* value for *Cox* proportional hazards regression analysis.

## 4 Discussion

In this study, we investigated the correlation between the expression of H3K27me3 and clinicopathologic features as well as prognosis of a relatively large cohort of 162 chordoma patients. Our data showed that the median age was 51 years, and the majority of patients were older than 50 years (57.4%) without gender predominance (M/F=1.5). The recurrence rate was approximately 38.8%, which was similar to the previous studies (5, 18–20). However, the tumor location of our study was mainly in skull base (57.4%), which was higher than previous reports (about 32%) (19, 20), for which one of the possible reasons might be due to more neurosurgical than orthopedic patients in our hospital. The 5-year OS for our cohort was approximately 75.9%, which is higher than the 5-year survival rate showed in the SEER database of 67.6% (4, 21), and we speculated that the higher OS was mainly related with the higher proportion of skull base location in our study, of which the 5-year OS was relatively higher (18, 22). Moreover, our findings suggested that patients age at onset, gender, and recurrence were not associated with outcomes (**Table 2**), which was consisted with literature reports (5, 8, 23). Due to the small number of patients with dedifferentiated chordoma and poorly differentiated chordoma in our series (n=6), outcomes estimates are not presented for these histologic subtypes.

Moreover, low expression of H3K27me3 was significantly correlated to unfavorable clinical features such as spine location, recurrence and poor differentiation (**Table 1**). The proportion of patients with low H3K27me3 expression in our collection was higher in the spine (36.2%, **Table 1**) than in the skull base (12.9%, **Table 1**), although in our results we did not find that patients with chordoma of the spine had poorer prognosis than patients with skull base location (**Tables 2**, **3**). We speculated that this reason may be related to the high proportion of cases that occurred in the skull base we collected. This factor may have contributed to our inconsistent conclusions with the literature.

To our knowledge, this is the first study to demonstrate that low expression of H3K27me3 was significantly associated with shorter OS and RFS of chordoma patients (**Tables 2**, **3**). These data demonstrated that H3K27me3 was a significant prognostic factors involved in chordoma progression and predicts unfavorable clinical outcomes. In addition, both dedifferentiated and poorly differentiated chordoma are known to have relatively poor prognosis, but the proportion of these two subtypes in chordoma is very low, so the reason for the poor overall prognosis of chordoma has to be found in conventional chordoma. In the present study, our cohort was enriched for conventional chordoma (96.3%), only 37 patients (37/156, 23.7%) with conventional chordoma had low H3K27me3 expression level (**Table 1**), which suggests that low expression of H3K27me3 remains a rare phenomenon in conventional chordoma. In dedifferentiated and poorly differentiated chordoma, H3K27me3 expression was high in all cases (**Figure 2**), suggesting the role of H3K27me3 in dedifferentiated and poorly differentiated chordoma may be different from that of conventional chordoma. A total of 18 patients in our study underwent postoperative radiotherapy (30 to 64 Gy equivalents), and we also underwent follow-up study by calling the discharged patients directly, however, some of them were unable to describe accurately whether received radiotherapy or not. To ensure the accuracy of data, we didn’t include the parameter of radiotherapy in our study. Nunna et al. has reported that neither radiotherapy nor chemotherapy were associated with OS. Therefore, the association of H3K27me3 with OS and RFS found in this study may not be related to radiotherapy (24).

For chordomas, wide en-bloc resection is not always possible, either because of the size or extent of the tumor or because such resection would lead to excessive morbidity (25).The limitation of this paper is that we did not provide accurate information about postoperative margins, because only 10 patients in our study reported clear postoperative negative margins (R0 margins), and none of the rest recorded margins. This may affect the accuracy of prognosis.

According to methylation profiling of tumor tissue, Jeffrey A. Zuccato et al. identified two stable tumor clusters by assessing pathways with genes, cluster 1 was termed as the Immune-infiltrated subtype and cluster 2 as the Cellular subtype. They also reported that the cluster 1 had a statistically significant poorer disease-specific survival than cluster 2 (26). Methylation of H3K27 is associated with fate determination during normal development and plays a notable role in dedifferentiation during disease. Therefore, as expected, it is related to many tumorigenesis processes and cancers (27). However, it does not always been proved to be of prognostic value when it absents. For example, in synovial sarcoma and oral squamous cell carcinoma, a high level of H3K27me3 was correlated with poor OS of patients (12, 28). Complete loss of H3K27me3 in some cancers, such as anaplastic meningioma, breast cancer and meningiomas has been associated with worse clinical outcome (13, 14, 29). These results indicate that our understanding of the clinical significance of H3K27me3 may not be comprehensive. As we all know, histones are an important part of eukaryotic chromatin and are responsible for the assembly and stability of chromatin (30). At the same time, a variety of chemical modifications on histones play an important role in epigenetic regulation, such as acetylation and methylation (31). H3K27me3 is the product of H3K27 trimethyl chemistry (32). Currently, there are two reasons for the reduction of H3K27me3. One reason is that H3K27M mutation blocks the methylation of K27 in the histone tail and reduces the overall level of H3K27me3 in cells (33), another reason is that in MPNSTs, when polycomb complex 2 (PRC2) is mutated, even without K27M mutation, histone H3 methylation is still affected, and H3K27me3 expression is lost (34). However, all of our cases showed positive immunohistochemistry for H3K27M, confirming that the mutation of H3K27M does not occur in chordoma. Therefore, our further work will focus on PRC2. Lucia Cottone et al. confirmed pharmacologic inhibition of the H3K27 demethylases JMJD3 and UTX can cause human chordoma cell death by inhibiting the expression of brachyury, which might be served as a therapeutic approach of chordoma (15).

In conclusion, the expression of H3K27me3 is associated with unfavorable clinical characteristics and poor prognosis of conventional chordoma patients. Although the role of H3K27me3 in tumorigenesis of chordoma remains unclear, H3K27me3 appears to be a significant prognostic factor in patients with conventional chordoma. In addition to serve as a potential prognostic marker, H3K27me3 could represent a novel target for therapeutic intervention in patients with conventional chordoma.

## Data availability statement

The raw data supporting the conclusions of this article will be made available by the authors, without undue reservation.

## Ethics statement

The studies involving human participants were reviewed and approved by Ethics Committees of Xi Jing Hospital. Written informed consent to participate in this study was provided by the participants’ legal guardian/next of kin.Written informed consent was obtained from the individual(s), and minor(s)’ legal guardian/next of kin, for the publication of any potentially identifiable images or data included in this article.

## Author contributions

LF and CX conceived and designed the experiment. JW, JFW, ZY and YL performed the experiments. YW, XL and ZW contributed the acquisition of data. JW, XL and CX analysed and interpreted the data. JW, JFW and ZY wrote and revised the manuscript. All authors contributed to the article and approved the submitted version.
